# Diabetes surpasses obesity as a risk factor for low serum testosterone level

**DOI:** 10.1186/s13098-024-01373-1

**Published:** 2024-06-28

**Authors:** Samir H. Assaad Khalil, Paresh Dandona, Nermin A. Osman, Ramy Samir Assaad, Basma Tayseer Abdalla Zaitoon, Amal Abdulaziz Almas, Noha Gaber Amin

**Affiliations:** 1 Department of Internal Medicine, Unit of Diabetes, Lipidology & Metabolism, Faculty of Medicine, Alexandria University, Alexandria, Egypt; 2 Department of Endocrinology, Faculty of Medicine, University of Buffalo and the State University of New York (SUNY), NY, USA; 3 Department of Biomedical Informatics and Medical Statistics, Medical Research Institute, Alexandria University, Alexandria, Egypt; 4 Data Science Institute, Imperial College London, London, UK; 5 Department of Chemical Pathology, Medical Research Institute, Alexandria University, Alexandria, Egypt; 6 Department of Internal Medicine, University of Nairobi, Nairobi, Kenya

**Keywords:** Testosterone, Lean, Obese, T2DM

## Abstract

**Background:**

Male obesity is one of the most associated factors with substandard testosterone levels. However, there is growing evidence linking low testosterone levels to insulin resistance and diabetic complications. We aimed to study the impact of diabetes mellitus on testosterone levels and to assess the correlation of various clinical and biochemical factors with hypogonadism.

**Subjects and methods:**

This case-control study was conducted on 160 adult males categorized into four equal groups (40 each); Group A: lean men with T2DM, Group B: obese with T2DM, Group C: lean with normal glycemic profile, Group D: obese with normal glycemic profile. Serum total testosterone (TT), SHBG and HbA1c have been measured. Free testosterone (cFT) and HOMA-IR were calculated.

**Results:**

A significant negative correlation of serum TT and cFTwith BMI (r -0.16, p 0.04/ r -0.26, *p* < 0.001, respectively) and with waist circumference (WC) (r -0.23, p 0.003 and r -0.3, *p* < 0.001, respectively). A significant decrease in TT and cFT in the diabetes group versus the non-diabetes one (*p* < 0.001 for both). TT level was significantly lower in the diabetic lean group than in the non-diabetic lean (*p* < 0.001), and even significantly lower than in the non-diabetic obese (*p* < 0.001). TT level in the diabetic obese group was lower than in the non-diabetic obese (*p* < 0.001). The same for cFT level, lower in the diabetic lean group than in non-diabetic lean (*p* < 0.001) and lower in the diabetic obese than in the non-diabetic obese (*p* < 0.001). Concomitant significant reduction in SHBG in the diabetes group (*p* < 0.001). Linear regression analysis revealed that TT significantly correlated with HOMA-IR. HOMA-IR with WC, age and the duration of diabetes correlated significantly with cFT. In our model, HOMA-IR and HbA1c accounted for approximately 51.3% of TT variability (adjusted R-squared 0.513).

**Conclusions:**

The impact of T2DM on serum testosterone levels was more significant than that of obesity. Our study showed a decrease in SHBG together with cFT among the diabetes group. Hypogonadism is significantly correlated to insulin resistance and poor glycemic control, which implies another perspective on the impact of suboptimal glycemic control on the development of hypogonadism.

## Introduction

Type 2 diabetes is a significant global public health burden. More than 536 million adults are estimated to have diabetes, and this is expected to increase in alignment with the obesity surge. Egypt is among the top 10 countries regarding the number of people with diabetes, exceeding a prevalence of 18.4% among the age group between 20 and 79 years, according to the IDF data [1]. Moreover, the age-adjusted prevalence of type 2 diabetes among adults in Alexandria was evaluated to be 16.8% [2].

Type 2 diabetes is commonly associated with obesity, insulin resistance, increased hepatic glucose output, and relative insulin insufficiency. People with type 2 diabetes often show abnormalities consistent with metabolic syndrome [3].

The association of low free testosterone levels in the presence of subnormal LH and FSH levels in men with type 2 diabetes was first reported in 2004 [4]. Several studies have confirmed the association of HH (Hypogonadotropic Hypogonadism) with type 2 diabetes, estimated to be prevalent among 25–40% of men with type 2 diabetes. Moreover, type 2 diabetes is included among the conditions associated with HH, according to the Endocrine Society, suggesting screening patients with type 2 diabetes for hypogonadism [5–8]. Although data show that total testosterone and free testosterone concentrations were inversely related to BMI and age, the presence of low T concentration was present among 25% of non-obese patients, confirming that HH was independent of obesity in many cases. In addition, HH was independent of the level of glycemic control and the duration of hyperglycemia [4].

Several studies reported low testosterone to be an independent risk factor for the development of type 2 diabetes [9–12]. Additionally, there is growing evidence linking low testosterone levels to the presence of insulin resistance status [13], an independent risk factor for the progression of diabetes-related complications, whether microvascular or macrovascular [14].

Epidemiologic and genetic evidence has also pointed to the possible impact of sex hormone-binding globulin (SHBG) in developing insulin resistance, metabolic syndrome, and type 2 DM [15–17]. Low serum SHBG levels are linked to the status of insulin resistance and hyperinsulinemia [15], indicating that SHBG could be a future risk factor predicting the occurrence of type 2 DM [18, 19].

The aim of this study was primarily to study the impact of diabetes mellitus on testosterone levels in men and to determine the clinical and biochemical correlates of hypogonadism. We aimed to compare testosterone levels (total and free) and sex hormone-binding globulin (SHBG) among patients with and without type 2 diabetes, in addition to predicting the significant factors affecting their levels among the studied population.

## Materials and methods

*Subjects and study design*: This case-control study was conducted by matching other confounding factors on adult male subjects attending the Diabetes and Metabolism Outpatient Clinic of Alexandria Main University Hospital as a part of their routine diabetes care. The study protocol received authorisation from the ethical committee. Informed written consent was obtained from all the subjects recruited for the study after the purpose and nature of the study were explained to them. The study was carried out between October 2021 and September 30, 2022. The study enrolled 160 male subjects aged between 27 and 45 years; the study subjects were randomly classified into four equal groups:

**Group A**: 40 lean men with T2DM: BMI < 25 kg/m².

**Group B**: 40 obese men with T2DM: BMI ≥ 30 kg/m².

**Group C**: 40 lean men with normal glycemic profile: BMI < 25 kg/m².

**Group D**: 40 obese men with normal glycemic profile: BMI ≥ 30 kg/m².

Group C and D subjects were chosen from accompaniers bringing in their DM relatives to the diabetes and metabolism outpatient clinic of Alexandria University Hospital. Patients with a known history of hypogonadism or a history of chronic debilitating diseases, such as severe hepatic impairment or renal failure, subjects with severe obstructive sleep apnea, and patients suffering from symptomatic depression were excluded from the study. We excluded participants with any previously diagnosed malignancy and those who have been prescribed medications for benign prostate hypertrophy or hypogonadism treatment that may impact testosterone levels such as SERMs (clomiphene, tamoxifen), aromatase inhibitors (e.g., letrozole), GnRH agonists, and 5-alpha reductase inhibitors (e.g., finasteride). Patients on insulin therapy were excluded from the study as this might interfere with HOMA- calculation.

We also excluded subjects who received testosterone therapy and over-the-counter health supplements comprising androgens, narcotics, or corticosteroids within the past three months.

*Demographic parameters and anthropometric measures*: Body weight and height were assessed, and body mass index (BMI) was calculated by dividing body weight in kilograms (Kg) by height in meters squared (m^2^). Waist circumference (WC) was measured at the end of expiration in a standing position midway between the lower rib margin and the superior iliac spine.

*Biochemical analysis*: Morning (before 10 am) blood samples were withdrawn from all participants after 8–10 h of fasting. Plain vacutainer samples were centrifuged, and the separated sera were used for the spectrophotometric measurement of fasting glucose and albumin. Serum insulin levels were estimated using a chemiluminescence technique with advanced acridinium ester technology (ADVIA Centaur immunoassay System – Siemens). Homeostasis model assessment 2 (HOMA-IR 2) was calculated for the estimation of insulin resistance of all participants, using the formula fasting glucose (mg/dL) X fasting insulin (mU/L) / 405. Serum total testosterone (TT) measurement was performed using a competitive solid phase enzyme-linked immunosorbent assay (ELISA - DRG Diagnostics Gmbh, Germany). The quantification of SHBG in serum samples was also performed using the sandwich technique solid-phase ELISA (DRG Diagnostics Gmbh, Germany). Free testosterone (cFT) was calculated from SHBG and testosterone using Vermeulen and colleagues’ method. Blood collected into EDTA vacutainers was used to directly determine glycated haemoglobin (HbA1c). This was performed by ion exchange high-performance liquid chromatography (HPLC – Tosoh Bioscience G8, Japan).

*Statistical analysis*: All statistical analyses were conducted using R version 4.2.1 with the following packages: tidyverse, dplyr, Nagpur, static, psych, complot, Hmisc, pROC, and randomForest. Categorical variables were described in terms of numbers and percentages. Shapiro’s normality test determines the mean and standard deviation usage for normally distributed data or the median (min-max) and interquartile range (IQR) for skewed data. Mann-Whitney tests defined diabetic and non-diabetic groups in various numerical data sets. For bivariate analysis between categorical variables, the Chi-square test was applied. We applied for the Monte-Carlo test in case of the Chi-Square test assumption violation. The Spearman Rho Rank Correlation test was conducted to determine the direction and strength of association between continuous variables.

The corrected F-welch ANOVA was used to investigate differences in Albumin and cFT among the four groups: A (DM and lean), B (DM and obese), C (lean), and D (obese). In contrast, the Kruskal-Wallis test was used to evaluate the rest of the study parameters. Three logistic regression models were conducted to predict the significant factors affecting cFT, TT, and SHBG. The significant *p*-value was set to less than 0.05.

## Results

**Fig. 1:**
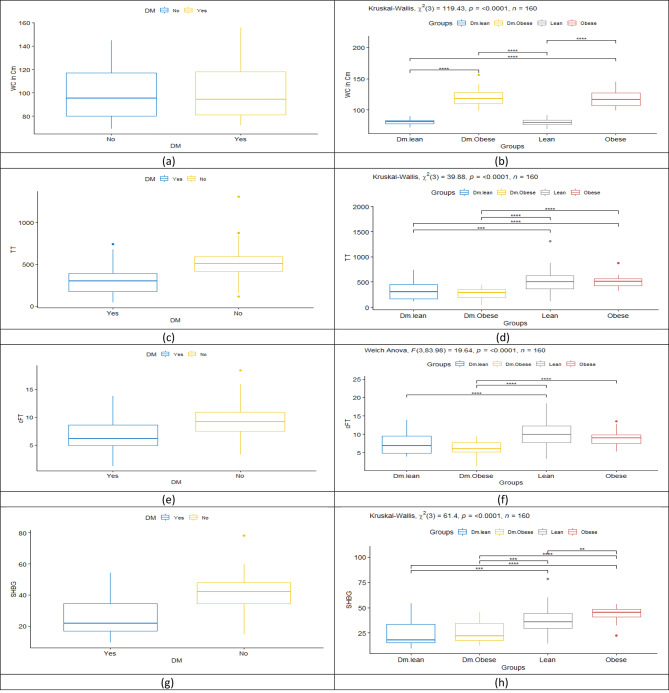
Box Plot Matrix illustrates the difference between the main diabetic and non-diabetic groups with the subgroup comparisons

Table 1 summarizes the general characteristics of the study population. The bivariate analysis between subjects with and without diabetes is shown in Table 2. The two groups had no significant difference regarding age, BMI, and WC. On the other hand, serum testosterone levels (total and free) and SHBG were statistically significantly higher in the non-diabetes group compared to the diabetes group. Figure 1 elucidates the difference regarding several studied parameters between the main diabetic and non-diabetic groups with the subgroup comparisons.

In the linear regression analysis, as shown in Table 3, total testosterone was significantly correlated with HOMA-IR and HbA1c, two important diabetes-related biomarkers. Based on the adjusted R-squared value of 0.513, these two variables account for approximately 51.3% of testosterone variability. A lower testosterone level is associated with higher insulin resistance and less glycemic control, as suggested by the negative coefficients for HOMA-IR and HbA1c. This model has the following equation: Total testosterone = 805.742–39.32 (HOMA-IR) − 44.977 (HbA1c).

**Fig. 2:**
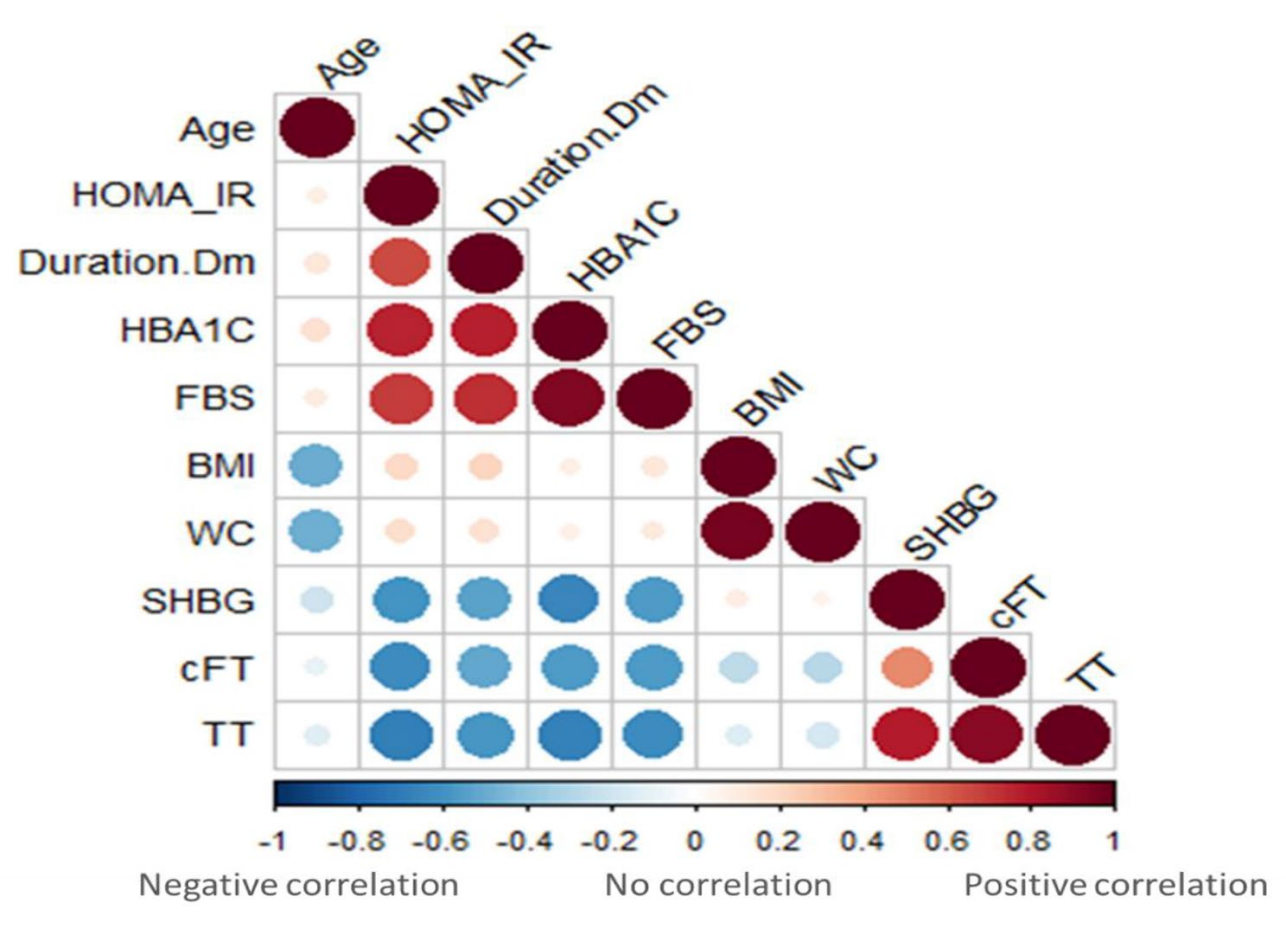
Correlation Matrix using Spearman Rho Rank test to determine the direction and strength of association between the main parameters

Several variables were associated with free testosterone as shown in figure 2, including duration of diabetes, waist circumference, age, and HOMA-IR. These variables can explain approximately 45.1% of free testosterone variability based on adjusted R-squared. Diabetes duration, WC, age, and IR were all associated with lower levels of free testosterone based on the negative coefficients. Using this model, Free testosterone would be calculated as follows: 19.76135 − 0.33104 (Duration of DM) − 0.03849 (Waist circumference) − 0.1329 (Age) − 0.72943 (HOMA-IR).

Regarding the SHBG, the linear regression analysis demonstrated a significant association between SHBG levels and several variables, including BMI, HbA1c, albumin, and total testosterone, as follows:

- BMI: SHBG levels are expected to increase by 0.35 units for each unit increase in BMI.
- HbA1c: For each unit increase in HbA1c, SHBG levels are expected to decrease by 3.43 units.
- Albumin: SHBG levels are expected to increase for each albumin unit increase by 5.38 units.
- Total testosterone: If the total testosterone level is less than 264, SHBG levels are expected to decrease by 10.49 units.

The adjusted R-squared value of 0.586 indicates approximately 58.6% of the variability in SHBG levels.

SHBG = 25.79 + 0.35(BMI) − 3.43(HbA1c) + 5.38(Albumin) − 10.49(TT (low)).

**Table 1:** Basic Characteristics of the studied population

Variable	Group	*N*	%
DM	Yes	80	50%
	No	80	50%
Study Groups	Diabetic and lean (I)	40	25%
	Diabetic and obese (II)	40	25%
	lean without DM (III)	40	25%
	obese without DM (IV)	40	25%
History of Smoking	Yes	83	51.88%
	No	77	48.13%
Age (Years)	median (min-max)	43(27–45)	
	IQR(q1-q3)	6(39–45)	
Duration of diabetes (Years)	Mean ± SD	1.15 ± 1.57	
	(Min-max)	(0–6)	
BMI (Kg/m^2^)	median (min-max)	27.45(21.5–41.9)	
	IQR(q1-q3)	7.55(24.07–31.62)	
Waist Circumference (cm)	median (min-max)	95.5(69–156)	
	IQR(q1-q3)	37.2(80.8–118)	

**Table 2:** Bivariate analysis between DM and non-DM groups using the Mann-Whitney test

variable		DM	non-DM	*p*
		*n* = 80	*n* = 80	
Age	median(min-max)	43(32–45)	42(27–45)	0.21
BMI	median(min-max)	27.45(21.5–41.9)	27.45(21.5–37)	0.67
WC	median(min-max)	94.5(72–156)	95.5(69–145)	0.82
cFT	median(min-max)	6.15(1.22–13.8)	9.22(3.29–18.4)	< 0.001*
TT	median(min-max)	297.5(40–740)	510(116–1310)	< 0.001*
SHBG	median(min-max)	21.7(9.3–54.2)	42.15(14.6–78.3)	< 0.001*
HOMA-IR	median(min-max)	3.79(0.9-10.76)	1.1(0.41–3.06)	< 0.001*
HbA1C	median(min-max)	8(6.9–11.3)	5.2(4.5-6)	< 0.001*

**Table 3:** Multiple linear regression analyses to identify the predicting factor(s) for the reduction of Total Testosterone, Free Testosterone, Serum HBG

model name:	Parameters	B-coefficient	*P*- value	*R*^2^, *R*^2^ adj	F _(df)_, model (*p*-value)
TT				0.52, 0.513	F_(2 ,157)_ = 84.98, < 0.001*
	(Intercept)	805.742	< 0.001*		
	HOMA_IR	-39.32	< 0.001*		
	HBA1C	-44.977	< 0.001*		
FT				0.465, 0.451	F_(4, 155)_ = 33.68, < 0.001*
	(Intercept)	19.76135	< 0.001*		
	Duration of DM	-0.33104	0.038*		
	WC	-0.03849	< 0.001*		
	Age	-0.1329	0.012*		
	HOMA_IR	-0.72943	< 0.001*		
SHBG				0.596, 0.586	F_(4, 155)_ = 57.23, < 0.001*
	(Intercept)	25.7877	0.004*		
	BMI	0.3523	0.017*		
	HBA1C	-3.428	< 0.001*		
	Albumin	5.3755	< 0.001*		
	TT (low)^a^	-10.4908	< 0.001*		

a: TT (low) means the serum TT is < 264.

## Discussion

In agreement with previous studies (20–22), the results from the current study demonstrated a significant negative correlation of total and free testosterone with BMI (r -0.16, p 0.04 and r -0.26, *p* < 0.001, respectively).

However, results have shown particularly as well a significant negative correlation between total and free testosterone with waist circumference (WC) (r -0.23, p 0.003 and r -0.3, *p* < 0.001, respectively). This finding was also reached in the Tromsø study [20], in which total testosterone was measured, and free testosterone was calculated in 1548 men and was analysed using anthropometric data. The age-adjusted correlation between WC and total and free testosterone was − 0.34 (*p* < 0.001) and − 0.09 (*p* < 0.001), respectively, which were stronger than that with BMI.

It is worth mentioning that central obesity and waist circumference as a marker for visceral fat have proved, without a doubt, to be independent risk factors for cardiovascular disease [21]. At the same time, lower levels of circulating testosterone have been reported to be positively associated with cardiovascular risk factors and atherosclerosis [22, 23]. With the background of this data, together with the reported association of testosterone and WC, an incriminated role for testosterone levels in men in the pathogenic link of central obesity and WC with cardiovascular risk can be proposed. Consistent with findings from adult males, the association between obesity and hypogonadism were also reported among young pubertal and post-pubertal males by Morgi et al., where testosterone levels were 40–50% lower than young males with normal BMI. Interestingly, following post-bariatric weight loss of one-third of the body weight among severely obese adolescents in a prospective multicentric study, a significant increase in testosterone levels decreased again on weight regain [24, 25].

However, the scope of our research was primarily to study the effect of diabetes mellitus on testosterone levels in men and to determine the clinical and biochemical parameters correlated to hypogonadism and the clinical predictors of low serum testosterone levels in men with type 2 diabetes. Therefore, the study participants were categorized into two groups: with and without diabetes. Each group consisted of 80 participants. The two groups included equal numbers of lean (*n* = 40, in each group) and obese (*n* = 40, in each group) participants. The two main groups (with and without DM) were statistically matched in terms of age (median 43 and 42 years respectively, p 0.21), BMI (median 27.45 and 27.45 Kg/m^2^ respectively, p 0.67), and WC (median 94.5 and 95.5 cm respectively, p 0.82).

Despite the nullification of the leading known variables affecting testosterone level (age, BMI and WC) between the two groups, there was a statistically significant decrease in the measured serum total testosterone in the diabetes group versus the non-diabetes one (median 297.5 and 510 ng/dL respectively, *p* < 0.001). The same has also been shown for the calculated free testosterone (cFT) (median 6.15 and 9.22 ng/dL, respectively, *p* < 0.001).

Male obesity is supposed to be one of the most commonly associated with substandard serum levels of testosterone. Despite that, on statistically comparing TT and cFT levels between the different subgroups in our study, TT level was significantly lower in the Diabetic Lean group than in the Non-Diabetic Lean (median 302 and 505 ng/dL, respectively, *p* < 0.001), and even significantly lower than in the Non-Diabetic Obese group (median 510 ng/dL, *p* < 0.001). In the same context, the TT level in the Diabetic Obese group was significantly lower than in the Non-Diabetic Obese group (median 284.5 and 510 ng/dL, respectively, *p* < 0.001). The same was true for the cFT level, which was significantly lower in the Diabetic Lean group than in Non-Diabetic Lean (*p* < 0.001) and was also significantly lower in the Diabetic Obese group than in the Non-Diabetic Obese group (*p* < 0.001).

These findings add to the accumulating evidence [26–30] that men with type 2 diabetes have a significantly greater prevalence of hypogonadism and impose diabetes per se to be one of the most associated conditions with decreased both total and free serum testosterone levels. This is slightly different from the meta-analysis conclusion by Grossmann et al. [27], reporting that the inverse association between testosterone and diabetes is stronger for total compared with free testosterone, which implies a role for SHBG given that total but not free testosterone changes in parallel with SHBG.

In our study, the decrease in the calculated free testosterone level was evident in the diabetes group in addition to the concomitant statistically significant reduction in the SHBG as well, in the diabetes group versus the non-diabetics (median 21.7 and 42.15 nmol/L respectively, *p* < 0.001). SHBG proved to relate to insulin resistance, an adiposity indicator [31]. There was a significant decrease in patients with diabetes compared to subjects without diabetes [32, 33]. Moreover, a lower level of SHBG was suggested as an independent predictor of incident type 2 diabetes mellitus in men [19]. One of the probable mechanisms by which elevated circulating SHBG protects from the development of type 2 DM is attributed to the regulation of fasting glycemia but without modification of the secretory function of insulin [17].

Dhindsa et al. suggested a hypogonadotropic mechanism for the low testosterone levels in diabetes, evidenced by the fact that LH and FSH levels were significantly lower in the 33% of hypogonadal patients of the 103 patients with diabetes enrolled in the study [4]. This aligns with the current study findings that total and free testosterone was significantly lower with diabetes and not peculiar TT as a function of lowered SHBG imposing a role for insulin resistance in type 2 diabetes as an implicated factor in the decreased total as well as free testosterone. These data were confirmed in our study, as we performed a linear regression analysis and emphasised that total testosterone was significantly correlated with HOMA-IR. Notably, the linear regression analysis of free testosterone also showed again that HOMA-IR with three other variables correlates significantly with cFT level, which was the WC (an important marker of insulin resistance), the age and the duration of diabetes.

The progressively evident causal relationship of insulin resistance with the decreased testosterone level seems bidirectional. This is believed as it has been reported that reduced levels of TT have been related to resistance to insulin and subsequent risk for T2DM development [34, 35]. It is still not clinically apparent to which extent low serum testosterone levels causally lead to type 2 diabetes. Theoretically, complex interactions among the hypothalamic–pituitary–gonadal axis, a status of insulin resistance, can give rise to glucose intolerance associated with ongoing low-grade inflammation and consequently increase the risk of cardiovascular disease [36]. Moreover, performed trials, though short-term, showed that testosterone supplementation in men may improve insulin sensitivity and reduce inflammation [37–39]. Furthermore, data from real-world registry reported that long-term testosterone treatment for patients with type 2 diabetes and hypogonadism was associated with improvement in glycemic control and insulin sensitivity. Interestingly, diabetes remission was achieved in one-third of the patients recruited in this 11-year data registry [40].

Another interesting finding in our model is that HOMA-IR and HbA1c- two significant correlating variables with TT- accounted for approximately 51.3% of TT variability (adjusted R-squared value of 0.513). This implies another perspective of the effect of glycemic control on hypogonadism and erectile dysfunction among males with diabetes, added to the known microvascular pathogenesis that involves a pro-inflammatory status that results in the decreased availability and activity of NO. In agreement with our results, Kim et al., involving Korean male patients with diabetes, reported that 34.9% of the 464 enrolled subjects had testosterone deficiency [41]. The testosterone deficiency group showed significantly higher mean fasting plasma glucose and HbA1c levels than the control group (*P* = 0.007 and 0.038, respectively). The results showed a significant negative correlation between fasting plasma glucose levels (*r*=-0.142, *P* = 0.002) and HbA1c values (*r*=-0.097, *P* = 0.040) with serum testosterone levels in men with diabetes.

This study encounters recruitment of patients attending Alexandria University Hospital which is a tertiary center receiving patients from four governments. It is an observational case-control unicentric study based on admission in the pre-determined study period; it is recommended to be conducted in a higher evidence base with a larger sample size. However, post hoc power was estimated by the end of the analysis to be 83%. The main study limitation is that the results were only valid among our involved population, so the conclusions may not be applied to other populations.

## Conclusion

This observational case-control study confirms that diabetes per se imposes a significant impact on both low total and low free testosterone, and SHBG. Unlike previous studies, our study investigated the levels of total testosterone, free testosterone and SHBG in both obese and lean patients with type 2 diabetes in comparison to healthy controls. Several factors – beyond BMI- were highly associated with low testosterone levels, mainly insulin resistance, visceral adiposity, poor glycemic control, and increased duration of diabetes. The impact of type 2 diabetes on serum testosterone levels is shown to be more significant than that of obesity. The significant correlation of hypogonadism to poor glycemic control implies another perspective on the impact of suboptimal glycemic control on hypogonadism complications of diabetes.

## Data Availability

No datasets were generated or analysed during the current study.

## Abbreviations

HbA1c: glycosylated hemoglobin
BMI: body mass index
